# Hemorrhagic Transformation (HT) and Symptomatic Intracerebral Hemorrhage (sICH) Risk Prediction Models for Postthrombolytic Hemorrhage in the Stroke Belt

**DOI:** 10.1155/2013/681673

**Published:** 2013

**Authors:** James E. Siegler, Muhammad Alvi, Amelia K. Boehme, Michael J. Lyerly, Karen C. Albright, Reza Bavarsad Shahripour, Pawan V. Rawal, Niren Kapoor, April Sisson, J. Thomas Houston, Anne W. Alexandrov, Sheryl Martin-Schild, Andrei V. Alexandrov

**Affiliations:** 1 Stroke Program, Department of Neurology, Tulane University School of Medicine, New Orleans, LA 70112, USA; 2 Stroke Program, Department of Neurology, School of Medicine, University of Alabama at Birmingham, RWUH 226M, 1720 2nd Avenue S, Birmingham, AL 35249, USA; 3 Department of Epidemiology, School of Public Health, University of Alabama at Birmingham, AL 35249, USA; 4 Health Services and Outcomes Research Center for Outcome and Effectiveness Research and Education (COERE), Birmingham, AL 35249, USA; 5 Center of Excellence in Comparative Effectiveness Research for Eliminating Disparities (CERED), Minority Health & Health Disparities Research Center (MHRC), Birmingham, AL 35249, USA; 6 School of Nursing, University of Alabama at Birmingham, AL 35249, USA

## Abstract

**Background:**

Symptomatic intracerebral hemorrhage (sICH) remains the most feared complication of intravenous tissue plasminogen activator (IV tPA) treatment. We aimed to investigate how previously validated scoring methodologies would perform in treated patients in two US Stroke Belt states.

**Methods and Results:**

We retrospectively reviewed consecutive patients from two centers in two Stroke Belt states who received IV tPA (2008–2011). We assessed the ability of three models to predict sICH. sICH was defined as a type 2 parenchymal hemorrhage with deterioration in National Institutes of Health Stroke Scale (NIHSS) score of ≥4 points or death. Among 457 IV tPA-treated patients, 19 (4.2%) had sICH (mean age 68, 26.3% Black, 63.2% female). The Cucchiara model was most predictive of sICH in the entire cohort (AUC: 0.6528) and most predictive of sICH among Blacks (OR = 6.03, 95% CI 1.07–34.1, *P* = 0.0422) when patients were dichotomized by score.

**Conclusions:**

In our small sample from the racially heterogeneous US Stroke Belt, the Cucchiara model outperformed the other models at predicting sICH. While predictive models should not be used to justify nontreatment with thrombolytics, those interested in understanding contributors to sICH may choose to use the Cucchiara model until a Stroke Belt model is developed for this region.

## 1. Background

Despite multiple acute stroke treatment trials, thrombolytic therapy with intravenous tissue plasminogen activator (IV tPA) remains the only FDA-approved acute treatment for ischemic stroke with proven long-term benefit [4, 5]. This treatment, while highly effective when administered in the appropriate setting, is not without risk. As many as 6% of patients treated with IV tPA for ischemic stroke were found to clinically deteriorate in the earliest randomized trial [5]; however current rates of symptomatic intracranial hemorrhage (sICH) are lower [6] and hemorrhagic transformation (HT) rates are around 9% [7]. These differences may be explained by differences in criteria used to define sICH [8].

Due to provider concern for sICH, early efforts at minimizing these adverse events focused on identifying sICH risk factors. Elevated serum glucose [9–11] or ferritin [12] on admission, history of diabetes mellitus [13], older age [10], greater baseline stroke severity [10, 14–16], and current tobacco use [14, 17] have all been identified as potential risk factors for sICH. In an effort to more accurately predict which patients are at the greatest risk of postthrombolytic hemorrhagic transformation (HT) [1, 18, 19] and/or sICH [2, 18–20], composite scores that integrate multiple demographic and clinical factors have been developed. To date, no score has been designed or tested specifically for ischemic stroke patients in the Stroke Belt of the United States. The Stroke Belt is an area of the southern US where the patient population has been repeatedly described as distinct from the remaining nationwide cohort [21–24], with higher mortality related to stroke, and IV tPA treatment rates can be as high as 30% [25].

The goals of this investigation were (1) to validate existing scoring methodologies [1–3] in predicting sICH in our cohort of ischemic stroke patients who live in the US Stroke Belt and (2) to compare the efficacy of these scoring methodologies in predicting risk of sICH for ischemic stroke patients in the US Stroke Belt.

## 2. Methods

We conducted a retrospective analysis of prospectively collected data using patients admitted from 2008 to 2011 to two academic stroke centers in two Stroke Belt states. Only patients treated with IV tPA were included. Patients treated with adjuvant intra-arterial thrombolysis were not excluded from the analysis. Trained neurologists collected imaging data retrospectively, blinded to the risk score variables. Select clinical, imaging, and laboratory data were used to calculate the following risk of sICH scores (1) intracranial hemorrhage risk score developed by Cucchiara et al. [1], (2) the safe implementation of treatments in stroke-sICH (SITS-sICH) risk score [2, 26], and (3) stroke prognostication using age and NIHSS (SPAN-100) score [3]. These scoring methodologies are summarized in Table 1. Given recent findings that ICD9 codes cannot accurately identify HT after ischemic stroke, HT was confirmed by vascular neurologists using 24-hour IV tPA follow-up noncontrast computed tomographic imaging of the head [27]. sICH was defined as a type 2 parenchymal hemorrhage with deterioration in National Institutes of Health Stroke Scale (NIHSS) score of ≥4 points or death [28]. Baseline characteristics between patients who did and did not experience sICH were compared using Pearson Chi Square and Wilcoxon Rank Sum tests. Binary logistic regression and receiver operating characteristic (ROC) curves were used to assess the ability of each scoring system to predict sICH. Scoring systems were tested in the entire population and then stratified by race. All tests were performed at the alpha = 0.05 significance level. This study was approved by our institutional IRB. The scoring systems were assessed by looking at the scores continuously, in four categories, and by dichotomizing the scores. The Cucchiara score is already categorized as a score of 0–4 whereas the SITS-sICH score was broken into low, average, moderate, and high risk based on the original design. The Cucchiara score was further dichotomized into <3 and ≥3, while the SITS-sICH score was further dichotomized into low/average versus moderate/high.

## 3. Results

Of the 457 ischemic stroke patients that met inclusion criteria,19 (4.2%) had sICH. All patients with sICH were included in the analysis. Patients who experienced sICH had higher admission serum glucose levels compared to those who did not experience sICH. Of note, a slightly larger proportion of patients with sICH group were diabetic (31.6% versus 26.8%, *P* = 0.6441), yet fewer were taking oral diabetes medications 5.6% versus 15.6%, *P* = 0.1631). Only 1 of the 19 (5.9%) patients with sICH reported actively using tobacco compared to 27.1% in those who did not experience sICH (Table 2).

**Figure 1:**
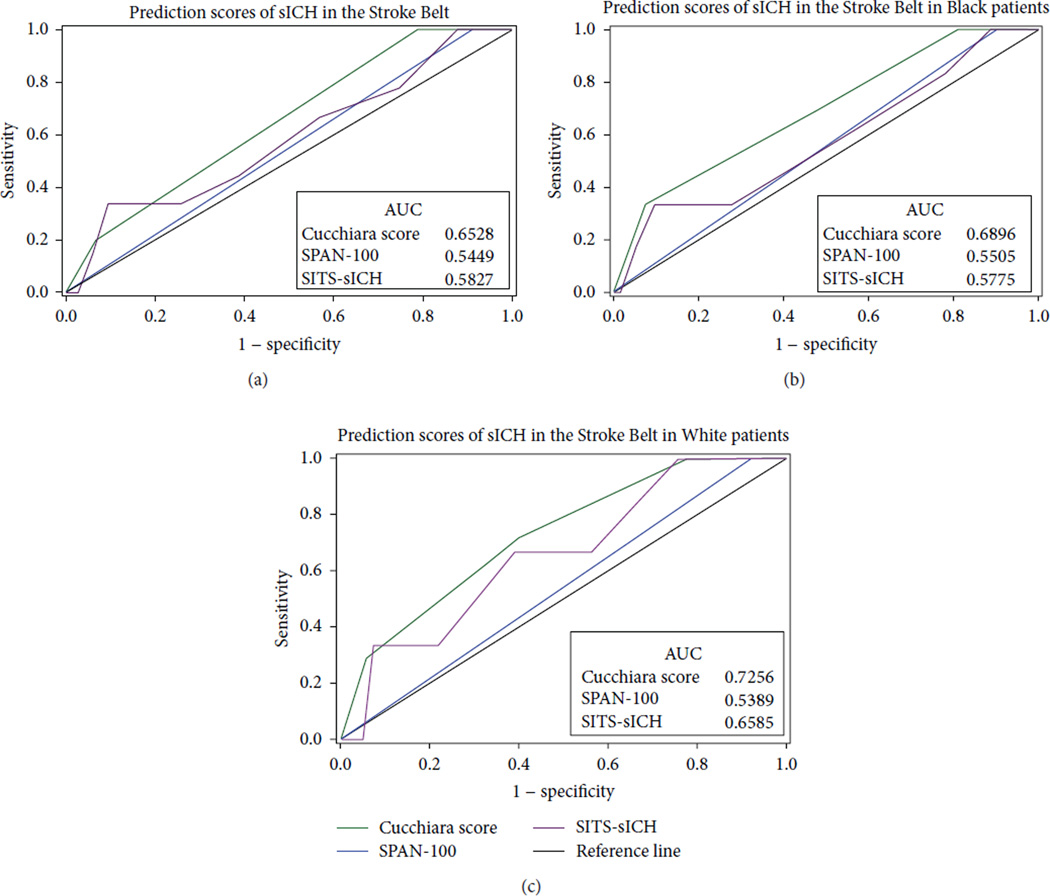
Receiver operator characteristic assessing ability of scores to predict sICH for all patients (a), Black patients (b), and White patients (c). sICH denotes symptomatic intracranial hemorrhage, AUC area under the curve, SPAN-100 stroke prognostication using age and National Institutes of Health Stroke Scale score, and SITS safe implementation of treatments in stroke.

The Cucchiara model, which was designed to predict HT, [1] was found to be most predictive of sICH (AUC: 0.6528) of the 3 scoring methods (Figure 1). When stratified by race, the Cucchiara score remained most predictive of sICH for both Blacks (AUC: 0.6896) and Whites (AUC: 0.7256) in our sample.

### 3.1. Four-Category Score Comparisons

**Figure 2:**
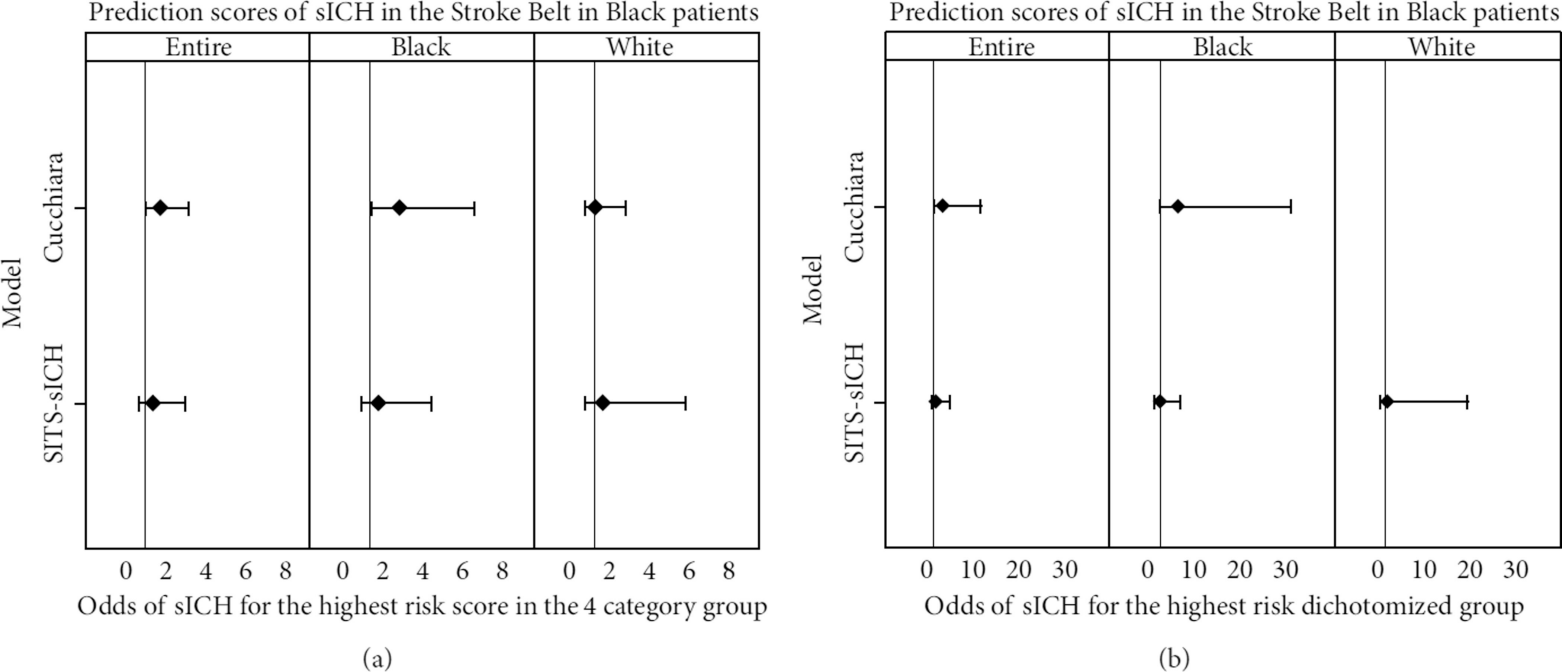
Odds of sICH according to selected scoring methodologies in our cohort of Stroke Belt patients using (a) 4-group and (b) dichotomized comparisons. sICH denotes symptomatic intracranial hemorrhage and SITS safe implementation of treatments in stroke.

As depicted in Table 3 and Figure 2, for patients classified as having a high risk Cucchiara score in the 4-category groups, the odds of having sICH were two times greater than any of the other groups (OR 2.02, 95% CI 1.08–3.81). On the other hand, in patients classified as having a high risk according to their 4-category SITS-sICH score, the odds of having sICH were only one and a half times that of those not in the other three groups (OR 1.51, 95% CI 0.63–3.63), but the association was not statistically significant. For Black patients in the high-risk 4-category Cucchiara score, the odds of having sICH were three times that of those in the other groups (OR 2.91, 95% CI 1.10–7.72).This is in contrast to patients who are classified as having a high risk according to their 4-category SITS-sICH score, where the odds of having sICH were more than one and a half times that of those in the other categories (OR 1.64, 95% CI 0.54–4.97), but the association was not statistically significant. In White patients with a high risk score according to their 4-category Cucchiara score, the odds of having sICH when compared to those not in the high risk group were similar (OR 1.02, 95% CI 0.34–3.02), while those classified high risk according to the 4-category SITS-sICH score had more than one and a half times the odds of sICH compared to those in the lower risk groups (OR 1.63, 95% CI 0.39–6.86), but the association was not statistically significant.

### 3.2. Dichotomized Score Comparisons

In patients categorized as having a high risk Cucchiara score in the dichotomized groups, the odds of having sICH were nearly three and a half times that of those not in the high risk group (OR 3.42, 95% CI 0.90–12.9), but the association was not statistically significant. On the other hand, patients categorized as having a high risk according to their dichotomized SITS-sICH score, the odds of having sICH were only 1.3 times that of those not in the high risk group (OR 1.26, 95% CI 0.33–4.85), but the association was not statistically significant. We were unable to calculate the odds of sICH in patients classified as high risk by the dichotomized SPAN-100 score, as none of our sICH patients had a SPAN-100 score of 100 or greater. In Black patients classified as high risk using the dichotomized Cucchiara score, the odds of having sICH were six times that of those not in the high risk group (OR 6.03, 95% CI 1.07–34.1). This is in contrast to patients classified as having a high risk according to their dichotomized SITS-sICH score, where the odds of having sICH were 1.2 times that of those not in the high risk group (OR 1.24, 95% CI 0.24–6.38), but the association was not statistically significant. In White patients, only the SITS-sICH score model converged, demonstrating that in White patients classified as high risk using the dichotomized SITS-sICH score, the odds of sICH were nearly two times that of those not in the high risk group (OR 1.78, 95% CI 0.14–21.92), but the association was not statistically significant.

**Figure 3:**
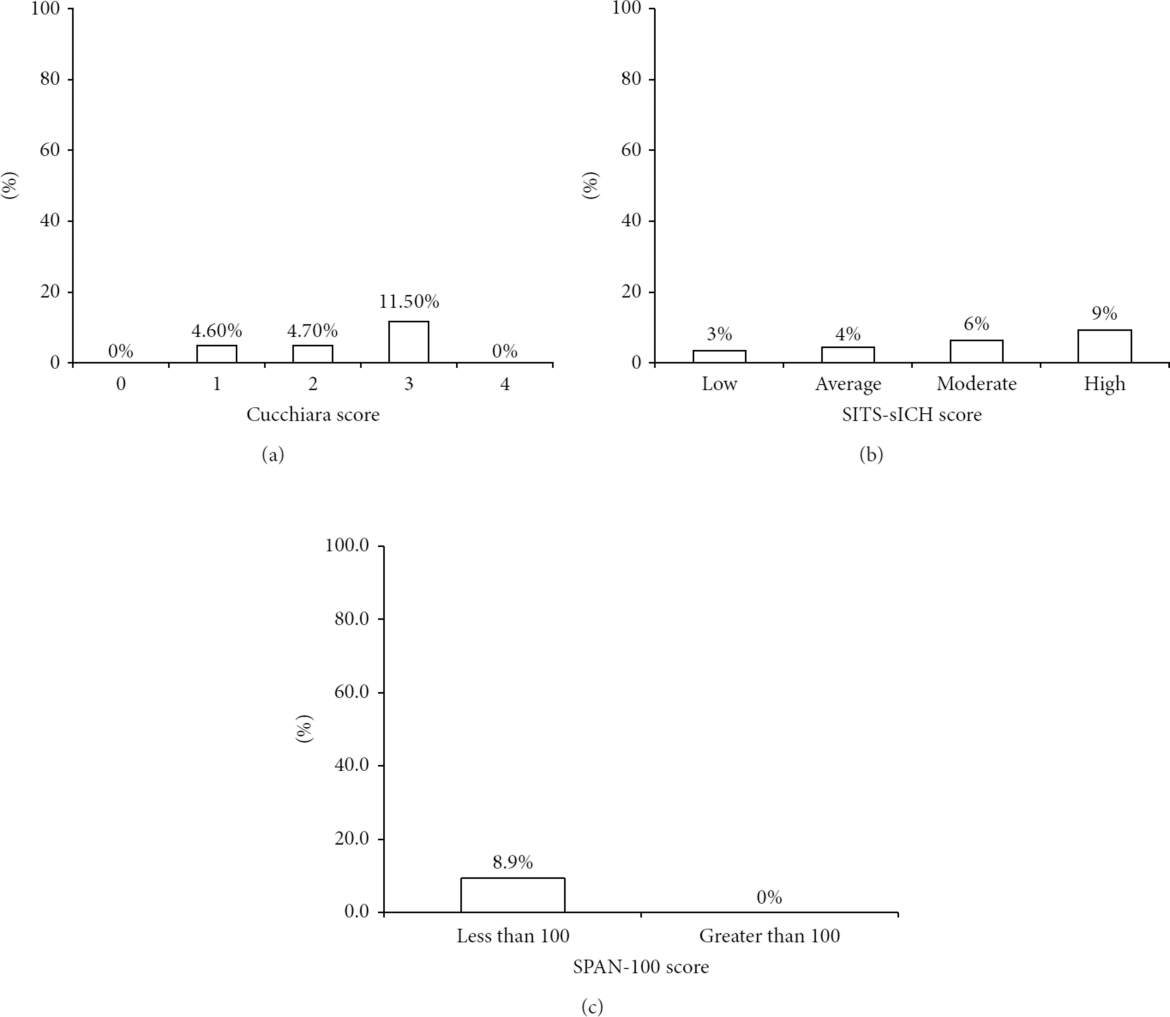
Percentage of patients with sICH according to clinical score. Values represent percentage of patients who experienced sICH according to score type and score value. In the SITS-sICH score, low corresponds to 0–2 points, average corresponds to 3–5 points, moderate corresponds to 6–8 points, and high corresponds to 9–12 points. sICH denotes symptomatic intracranial hemorrhage, SITS safe implementation of treatments in stroke, and SPAN-100 stroke prognostication using age and National Institutes of Health Stroke Scale score.

Figure 3 depicts the percentage of patients who experienced sICH according to selected risk scores. No patient with the lowest Cucchiara score experienced sICH. No patient within our sample was classified as having a Cucchiara score of 4. sICH rate was similar in patients with Cucchiara scores of 1 and 2, but 11.5% in patients with scores of 3. For the SITS-sICH score, rate of sICH was lowest in the low-score group and highest in the high-score group, but average and moderate scores had disproportionate rates of sICH. As previously stated, there were no patients with sICH with a SPAN-100 score of greater than 100 in our sample.

## 4. Discussion

While previously established scoring tools for predicting HT or sICH after thrombolysis have been validated in other populations [29], we did not find them to be generalizable to our sample of Stroke Belt patients. Using the data elements and thresholds described by the original investigators, we found the Cucchiara model to have the best external validity in predicting sICH after administration of thrombolysis, particularly when there is a significant Black population. The SITS-sICH model, on the other hand, was more effective at predicting sICH among Whites. Lastly, the SPAN-100 model, while incorporating baseline data elements similar to the other models, was not effective at predicting sICH in our population since none of the SPAN-100 positive patients experienced sICH.

Both the Cucchiara and SITS-sICH models use older age, greater baseline NIHSS scores, and higher serum glucose levels as components of their scoring systems; however, these two models may differ subtly at predicting HT or sICH due to the differential weight placed on components of the models (e.g., Cucchiara 25% of available points for hyperglycemia versus SITS-MOST 16.7% of available points) and the distinct thresholds used for their common components (e.g., Cucchiara baseline NIHSS >10 versus SITS-sICH baseline NIHSS 7–12 or >12). Furthermore, while the SITS-sICH score was designed to predict sICH, the Cucchiara model was designed to predict HT. It is possible that the Cucchiara model’s superior ability to predict sICH in Blacks and the SITS-sICH model’s ability to best predict sICH in Whites in both the 4-group and dichotomized analysis is a function of the underlying population used to create each scoring system. It is not surprising that SPAN-100 was outperformed by the other models, given that it was created not for the best performance but for use as a simple, easy-to-remember tool.

It is also important to distinguish which type of model can be most efficiently utilized in a given hospital system. While the SPAN-100model [3] may be calculated rapidly and with ease, it does not take into account factors previously shown to effect HT, such as serum glucose [9–11]. The SITS-sICH model [2] is much more encompassing but may be perceived as burdensome given the data points required. Given the known limitations of scoring systems of this nature, each center may benefit from determining the scoring method that most accurately predicts sICH in their patient population. While predictive models should not be used to justify nontreatment with IV tPA, those interested in understanding contributors to sICH after controlling for hypertension protocol violations and improper patient selection may choose to use the Cucchiara model until a Stroke Belt model is developed for this region.

The limitations of our study warrant discussion. Performing a retrospective analysis of data collected both prospectively and retrospectively limited the availability of data points and prohibited comparisons with other sICH predictive models such as the hemorrhage after thrombolysis (HAT) score [18], the iScore [19], and others that take brain imaging into consideration [20, 30]. Our small sample size limited our ability to attain precise estimates, while the small number of cases of sICH may have limited our ability to detect existing differences between scoring systems.

Despite its limitations, our study is unique in that it attempts to test the external validity of three sICH prediction scores by examining their performance in a sample taken from the Stroke Belt. Our results suggest that the existing scoring systems may need recalibration to work on racially diverse populations that inherently carry higher rates of diabetes and hypertension which are often poorly controlled. Additional study in larger, more diverse samples of IV tPA-treated patients is warranted.

## Figures and Tables

**Table 1:** Risk scoring methodologies for hemorrhagic transformation or symptomatic intracerebral hemorrhage following thrombolytic therapy.

Investigator	Score title	Primary outcome measure	Points awarded	Conclusions
Cucchiara et al. [1]	None	HT	4-point score with 1 point allotted for age >60 baseline NIHSS >10 baseline serum glucose >8.325mmol/L (150mg/dL) baseline platelet count <150,000/mm^3^	Proportion of patients with HT 0 points: 2.6% 1 point: 9.7% 2 points: 15.1% 3 or 4 points: 37.9% (AUC 0.69)
Mazya et al. [2]	SITS-sICH risk score	sICH	12-point score with 1 point allotted for baseline NIHSS 7–12 baseline systolic blood pressure ≥146mmHg age ≥72 years body weight ≥95 kg stroke onset to treatment time ≥180 min history of hypertension 2 points allotted for baseline NIHSS >12 baseline serum glucose ≥180mg/dL current use of aspirin (3 points if current use of combined aspirin and clopidogrel)	Proportion of patients with sICH 0 points: 0.2% ≥10 points: 14.3% (AUC 0.70)
Saposnik et al. [3]	SPAN-100	HT	Composite score considered positive if age + baseline NIHSS ≥100	Proportion of patients with HT SPAN-100 positive group 42% SPAN-100 negative group 12% (*P* < 0.001)

Table is not comprehensive. HT denotes hemorrhagic transformation; NIHSS: National Institutes of Health Stroke Scale; sICH: symptomatic intracerebral hemorrhage; CT: computed tomography of the head; MCA: middle cerebral artery; SITS: safe implementation of treatments in stroke; SPAN: stroke prognostication using age and National Institutes of Health Stroke Scale score.

**Table 2:** Comparision of patients who experienced sICH to those without sICH.

	No sICH (*N* = 438)	sICH (*N* = 19)	*P* value
Age, median y (range)	66 (20–99)	68 (40–89)	0.3117
Race, no. Black (%)	100 (22.8%)	5 (26.3%)	0.2538
Gender, no. female (%)	197 (45.5%)	12 (63.2%)	0.1307
Arrived within 3 hours, no. (%)	267 (63.1%)	10 (58.8%)	0.7191
Intra-arterial thrombolysis, no. (%)	15 (3.4%)	2 (10.5%)	0.3260
Past medical history, no. (%)			
Hyperlipidemia	148 (34.0%)	7 (36.8%)	0.7994
Hypertension	319 (73.2%)	15 (78.9%)	0.5766
Congestive heart failure	49 (11.2%)	1 (5.3%)	0.257
Atrial Fibrillation	66 (15.2%)	3 (15.8%)	0.5722
Diabetes	117 (26.8%)	6 (31.6%)	0.6441
Active smoker	118 (27.1%)	1 (5.9%)	0.0317
Laboratory values			
Admission glucose, median mg/dL (range)	115 (65–663)	153 (78–536)	0.0009
Admission platelet <150.000 (%)	6 (1.7%)	0 (0.0%)	0.7727
INR, median (range)	1.1 (0.7–2.3)	1.1 (0.8–2.5)	0.5794
Home medications			
Aspirin	136 (56.2%)	5 (38.5%)	0.1049
Clopidogrel	65 (26.9%)	3 (23.1%)	0.2492
Aspirin + clopidogrel	33 (15.7%)	2 (16.7%)	0.3036
Antihypertensive	220 (50.2%)	8 (44.4%)	0.6305
Oral diabetic medication	68 (15.6%)	1 (5.6%)	0.1631
Physical exam			
Admission systolic blood pressure, median mmHg (range)	157 (82–265)	169 (100–260)	0.3035
Admission diastolic blood pressure, median mmHg (range)	87 (45–187)	91 (44–126)	0.8446
Admission NIHSS, median (range)	9 (0–32)	10 (0–20)	0.5245

sICH denotes symptomatic intracranial hemorrhage; INR: international normalized ratio; and NIHSS: National Institutes of Health Stroke Scale score.

**Table 3:** Odds of sICH according to selected scoring methodologies in our cohort of Stroke Belt patients.

	OR	Odds of sICH 95% CI	*P* value
Entire population			
Cucchiara score			
4 groups (1, 2, 3, 4)	2.02	1.08–3.81	0.0287
2 groups (>=3)	3.42	0.90–12.9	0.0706
SITS-sICH			
4 groups (0–2, 3–5, 6–8, 9–12)	1.51	0.63–3.63	0.3593
2 groups (>=6)	1.26	0.33–4.85	0.7364
SPAN-100	n/a	n/a	n/a
Black patients			
Cucchiara score			
4 groups (1, 2, 3, 4)	2.91	1.10–7.72	0.0318
2 groups (>=3)	6.03	1.07–34.1	0.0422
SITS-sICH			
4 groups (0–2, 3–5, 6–8, 9–12)	1.64	0.54–4.97	0.3792
2 groups (>=6)	1.24	0.24–6.38	0.8009
SPAN-100	n/a	n/a	n/a
White patients			
Cucchiara score			
4 groups (1, 2, 3, 4)	1.02	0.34–3.02	0.9730
2 groups (>=3)	n/a	n/a	n/a
SITS-sICH			
4 groups (0–2, 3–5, 6–8, 9–12)	1.63	0.39–6.86	0.5049
2 groups (>=6)	1.78	0.14–21.92	0.6535
SPAN-100	n/a	n/a	n/a

SPAN-100 model was unable to converge in the present population. sICH denotes symptomatic intracranial hemorrhage; OR: odds ratio; CI: confidence interval; SITS: safe implementation of treatments in stroke; SPAN-100: stroke prognostication using age and National Institutes of Health Stroke Scale score.
